# Correlation Between Reduced Daily Living Competence and the Risk of Postoperative Delirium in Orthopedics and Trauma Surgery

**DOI:** 10.3390/jcm13226722

**Published:** 2024-11-08

**Authors:** Louisa Katharina Rahm, Henriette Louise Moellmann, Carla Stenmanns, Erik Schiffner, Joachim Windolf, Helmut Frohnhofen, David Latz

**Affiliations:** 1 Medical Faculty, Heinrich-Heine-University Düsseldorf, Universitätsstraße 1, 40225 Düsseldorf, Germany; louisakatharina.rahm@med.uni-duesseldorf.de; 2 Cranio-and-Maxillo Facial Surgery, University Hospital Düsseldorf, Moorenstraße 5, 40225 Düsseldorf, Germany; 3 Orthopedics and Trauma Surgery, University Hospital Düsseldorf, Moorenstraße 5, 40225 Düsseldorf, Germanyerik.schiffner@med.uni-duesseldorf.de (E.S.); joachim.windolf@med.uni-duesseldorf.de (J.W.); david.latz@med.uni-duesseldorf.de (D.L.); 4 Department of Medicine, Faculty of Health, University Witten-Herdecke, 58448 Witten, Germany

**Keywords:** postoperative delirium (POD), non-withdrawal delirium, activities of daily living (ADL), geriatric assessment, trauma surgery, orthopedic surgery

## Abstract

**Background/Objectives:** Postoperative delirium is a prevalent and serious complication among elderly patients following surgical procedures. Prior research indicates that reduced competence in daily living, as evidenced by limitations in performing Activities of Daily Living (ADL), is directly associated with reduced patient mobility. This study aimed to investigate the potential role of preoperative mobility as a risk factor for the development of postoperative delirium. **Methods:** To assess preoperative mobility, a comprehensive geriatric evaluation of daily living competence was conducted. This included the Katz Index of Independence in ADL, which assessed basic daily activities over the preceding 14 days, and the Instrumental Activities of Daily Living Scale (IADL). Postoperatively, delirium monitoring was performed twice daily for seven days using validated delirium screening tools, including the Nursing Delirium Screening Scale, the Confusion Assessment Method, and the 4ATest. **Results:** A significant correlation was observed between the incidence of delirium and the IADL scores in all patients, with *p* < 0.001 for men and *p* = 0.001 for women. Among emergency patients, the Katz Index scores significantly differed between those who developed delirium and those who did not (*p* = 0.015). Additionally, a significant correlation was found between the Katz Index and the incidence of delirium in both groups (*p* < 0.001). **Conclusions:** The findings of this study emphasize the necessity of preoperative geriatric assessment using tools such as the Katz Index or IADL to identify patients at risk of delirium. The results confirm the importance of enhanced postoperative monitoring for potential delirium. For elective patients, prehabilitation should be considered when reduced daily living competence is identified. For emergency patients, immediate postoperative interventions, including intensive mobilization and orthogeriatric co-management, are recommended.

## 1. Introduction

Postoperative delirium is an acute state of confusion characterized by disturbances in consciousness, attention, perception, and cognition, as defined by the Diagnostic and Statistical Manual of Mental Disorders, Fifth Edition (DSM-5) [1]. This condition is particularly prevalent in patients over 70 years of age following surgical procedures under general anesthesia [2,3]. The ongoing demographic shift, coupled with an increase in age-related trauma both in Germany and globally, presents new multidisciplinary challenges for the healthcare system, especially concerning the perioperative management of elderly, comorbid patients [4,5,6]. The rising number of geriatric patients undergoing orthopedic surgeries has led to an increased incidence of postoperative delirium, highlighting its growing significance in clinical practice [7]. Consequently, identifying and mitigating the risk factors for postoperative delirium is critical for improving patient safety and clinical outcomes [8].

The risk factors for postoperative delirium are versatile and mainly not validated yet. Nonetheless, they can be divided into two categories: predisposing and precipitating factors [9,10]. Predisposing factors are those that increase patient susceptibility to postoperative delirium, regardless of the type of surgical procedure. These include advanced age, cognitive impairment, frailty, depression, polypharmacy, and sensory deficits [11]. Precipitating factors are those that trigger or exacerbate delirium, depending on the type and course of the surgical procedure [12]. These include pain, infection, medications, fluid and electrolyte disturbances, type of anesthesia, blood loss, hypoxia, and immobilization [13].

Previous studies have suggested a correlation between mobility, cognitive competence, mental well-being, pre-existing conditions, comorbidities, preoperative nutritional status, and the risk of delirium [14,15]. The interaction of these factors significantly influences a patient’s competence in Activities of Daily Living (ADL), which emerges as a major predisposing but adjustable factor for postoperative delirium. Validated tools such as the Katz Index of Independence in Activities of Daily Living (Katz Index) and the Lawton-Brody Instrumental Activities of Daily Living scale (IADL) are commonly used to assess patients’ independence and functional limitations [16,17]. 

To validate the correlation between impaired mobility and an increased risk of postoperative delirium, it is essential to assess patients’ competence in performing ADL, which serves as a surrogate marker for mobility and frailty status [18].

This study investigates the relationship between mobility and postoperative delirium, with a focus on reduced daily living competence as a potential risk factor.

## 2. Materials and Methods 

### 2.1. Study Population 

This study utilized an interdisciplinary dataset encompassing patients from the departments of orthopedics and trauma surgery, maxillofacial surgery, vascular surgery, and general surgery, collected between August 2022 and August 2023. Inclusion criteria for the study were an age of at least 70 years and no preoperatively diagnosed dementia. Importantly, none of the patients included in the study presented with delirium at the time of admission.

To investigate the potential impact of varying daily living competence between patient groups, the cohort was divided into two groups: those undergoing emergency surgery and those undergoing elective surgery.

### 2.2. Screening Tools

**Figure 1:**
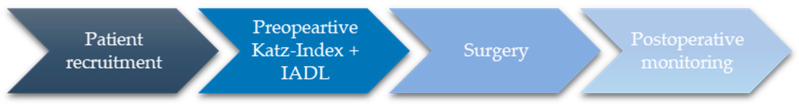
Overview of the study procedure.

A comprehensive geriatric assessment was conducted upon admission to evaluate the patients’ competence in performing ADL. The preoperative assessment captured variables such as age, gender, and ADL using the Katz Index and IADL [19]. For elective surgery patients, this assessment was conducted upon hospital admission, while for emergency surgery patients, it was performed immediately before surgery. First, Katz Index in ADL and the IADL score according to Lawton and Brody are gathered. As illustrated in Figure 1, the Katz Index and IADL were assessed during the preoperative admission process. A Katz Index score of 6 indicates complete independence, while scores between 3 and 5 suggest moderate functional ability. Scores ranging from 0 to 2 indicate complete dependence [20]. It includes bathing, dressing, toileting, transferring, continence, and feeding [19]. Another screening tool used to assess daily living skills is the IADL scale according to Lawton and Brody. It requires assessing whether the patient can independently perform tasks such as bathing, dressing, housework, going upstairs, going out alone, going shopping, taking care of finances, taking medications, and preparing meals [21]. The IADL scale, which is a validated screening tool for evaluating daily living skills, was adjusted based on gender: women were assessed on eight items, whereas men were assessed on five items, excluding cooking, housekeeping, and laundry.

No further patient interactions were conducted until the day of the elective procedure. 

Postoperative monitoring commenced on the first day after surgery. Postoperatively, delirium screening was performed using validated screening tools, including the Nursing Delirium Screening Scale (Nu-DESC), which was routinely administered in the recovery room by anesthesiologists. The nursing delirium screening scale (Nu-DESC) is collected through the interaction of the nursing staff with patients. For this purpose, there are five characteristics to be tested. These include disorientation, inappropriate behavior, inappropriate behavior, inappropriate communication, illusion/hallucination, and psychomotor retardation [22]. Additionally, the Confusion Assessment Method (CAM) was employed from the time of transfer to the ward. Postoperative monitoring was conducted twice daily on the ward for seven days using the Nu-DESC, CAM, and the 4AT (Assessment Test for Delirium & Cognitive Impairment). The assessment test for delirium and cognitive impairment (4AT) differentiates between four categories and examines the patient’s alertness and orientation. The combination of CAM and 4AT enabled a multifactorial assessment of the patient’s status, facilitating the early and precise detection of postoperative delirium. The Nu-DESC was administered through interactions between nursing staff and patients, assessing five specific characteristics [22]. The CAM was also utilized during daily ward rounds, while the 4AT assessed four categories related to patient alertness and orientation [23]. The seven-day postoperative delirium screening was concluded with a final assessment of mobility and physical condition using the Katz Index prior to discharge [3,24].

### 2.3. Ethics

This study was approved by the ethics committee of the Medical Faculty at Heinrich Heine University in Düsseldorf (Study No.: 2022-1810). All participants provided written informed consent. The research project was registered in a publicly accessible database (Deutsches Register Klinischer Studien, DRKS-ID: DRKS00028614) in accordance with DvH2013, § 35, prior to the recruitment of the first patient.

### 2.4. Statistical Analysis

The measured values and clinical data were statistically analyzed using jamovi (version 1.6.9, (Computer Software; retrieved from https//www.jamovi.org, accessed on 19 March 2022, Sydney, Australia). Mean differences were tested with independent *t*-tests (t) when significant outliers identified with boxplots were excluded, normality of the dependent variable was tested with the Shapiro–Wilk test, and homoscedasticity was tested with the Levene test. Differences in mean values for non-normally distributed dependent variables or a lack of homogeneity of variance were analyzed using the Mann–Whitney U test (U) or the Yuen test. A contingency table was created for categorical variables. The chi-square test was used to test correlations between categorical variables. It indicates the probability with which the observations of the study can be transferred to the population. A *p*-value of <0.05 was defined as significant, a value of <0.01 as very significant, and a value of <0.001 as highly significant. A significance level of *p* > 0.05 was defined for the hypothesis test.

## 3. Results

### 3.1. Emergency vs. Elective Surgery

#### Descriptive Statistics

The results presented in this study are based on data from 168 orthopedic and trauma surgery patients. Of these, data from 157 patients were available for analysis. The final dataset included 157 patients, consisting of 110 women (70.06%) and 47 men (29.94%), with a mean age of 79.0 ± 5.7 years. These patients underwent surgery between August 2022 and August 2023. To examine the potential influence of varying mobility scores, patients were categorized into two groups: those undergoing emergency surgery (*n* = 111) and those undergoing elective surgery (*n* = 46).

An overview of the patients’ personal and clinical data, as well as outcome parameters, is provided in Table 1, which also presents descriptive statistics for various parameters in both emergency and elective patients, including the results of the Shapiro–Wilk test for normal distribution.

**Figure 2:**
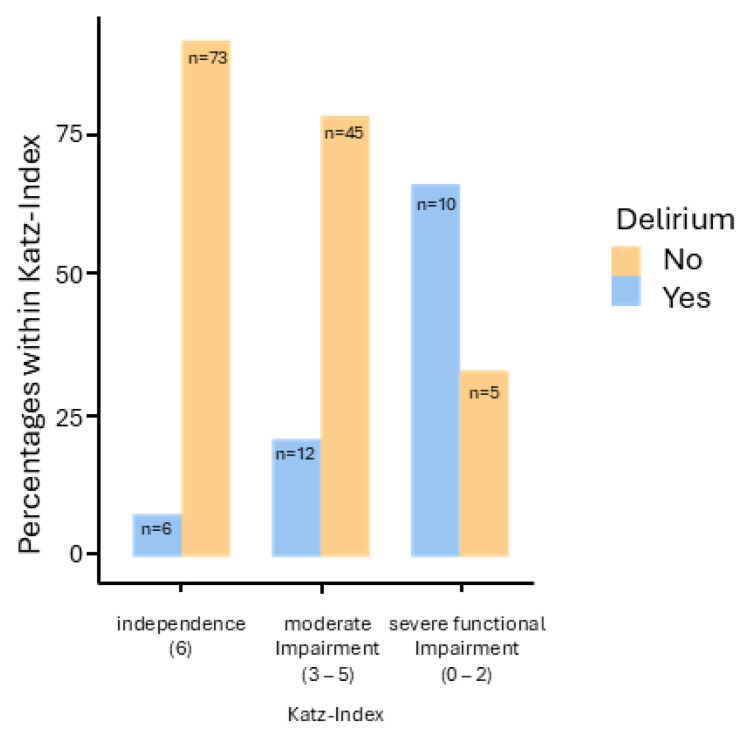
Presentation of patients with and without delirium within the groups in the Katz Index (independence, moderate impairment, and severe functional impairment).

The correlation between functional impairment and the occurrence of delirium was statistically significant, X^2^(2) = 29.5, *p* < 0.001, with a Cramer’s V of 0.442. The incidence of delirium was 7.6% (*n* = 6) in patients without impairment (*n* = 79), 21.2% (*n* = 12) in patients with moderate impairment (*n* = 57), and 66.7% (*n* = 10) in patients with severe functional impairment (*n* = 15) (see Figure 2).

**Figure 3:**
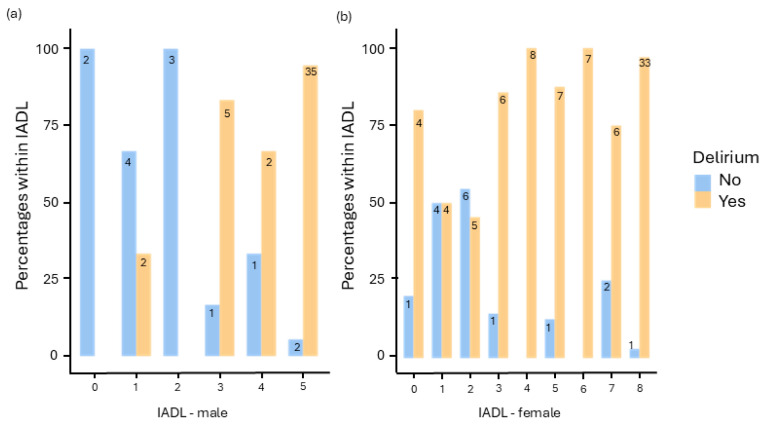
Presentation of male (**a**) and female (**b**) patients with and without delirium depending on the score in the IADL.

A significant correlation was found between the occurrence of delirium and the IADL score in men, X^2^(5) = 30.2, *p* < 0.001, Cramer’s V = 0.727 (see Figure 3a). Similarly, for women, the correlation was significant, X^2^(8) = 25.9, *p* = 0.001, Cramer’s V = 0.520 (see Figure 3b). 

### 3.2. Emergency Surgery

To assess the impact of potential limitations in ADL on the incidence of delirium, a binomial logistic regression (BLR) was conducted. The BLR model was significant, Χ^2^(3) = 10.0, *p* = 0.018, with a Nagelkerke’s R^2^ of 0.128. The model’s accuracy was 78.2%, with a specificity of 5.26% and a sensitivity of 95.1%. With a coefficient of determination of R^2^ = 0.128, a sample size of 103, and a significance level of α = 0.05, the statistical power for two predictors would be 1 − β = 0.93913. Statistical power indicates the probability of committing an error of the second kind. Here, the probability of committing a second type of error would be 6.09%. In 6.09% of cases, the test would not indicate significance, even if it were actually significant [25]. However, none of the examined variables (Katz Index *p* = 0.293, IADL *p* = 0.072) was statistically significant. Detailed model coefficients and odds ratios are presented in Table 2.

**Figure 4:**
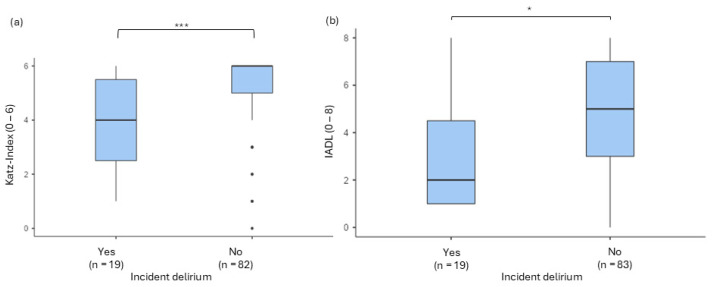
Comparison of (**a**) Katz Index and (**b**) IADL related to delirium incidence in emergency surgery (* *p* < 0.05; *** *p* < 0.001, dots = outliers).

A comparison of the preoperative mean values (Katz Index (*n* = 101) and IADL (*n* = 102)) between patients with and without delirium revealed significant differences. The IADL scores (delirium IQR 3.50, 95% CI [1.91; 3.98], no delirium IQR 4.00, 95% CI [4.45; 5.48]) showed a significant difference between the two groups, U = 412, *p* = 0.001, and r = 0.478. The Katz Index scores also differed significantly between groups (delirium IQR 3.00, 95% CI [3.12; 4.77], no delirium IQR 1.00, 95% CI [4.86; 5.43]), as indicated by the Yuen *t*-test, Ty (12.7) = 2.81, *p* = 0.015, and ξ^2^ = 0.555 (see Figure 4).

### 3.3. Elective Surgery

To further demonstrate the effect of ADL limitations on delirium incidence, another binomial logistic regression was performed. This model was also significant, Χ^2^(3) = 10.1, *p* = 0.006, with a Nagelkerke’s R^2^ of 0.453. The model achieved 95.3% accuracy, with a specificity of 50.0%, and sensitivity at 100.0%. With a coefficient of determination of R^2^ = 0.453, a sample size of 43, and a significance level of α = 0.05, the statistical power for two predictors would be 1 − β = 0.99969. The statistical power indicates the probability of committing an error of the second kind. Here, the probability of committing a second type of error would be 0.03%. In 0.03% of cases, the test would not indicate significance, even if it were actually significant [25]. Despite this, none of the variables (Katz Index *p* = 1.000, IADL *p* = 0.999) reached statistical significance. The detailed model coefficients and odds ratios are presented in Table 3.

**Figure 5:**
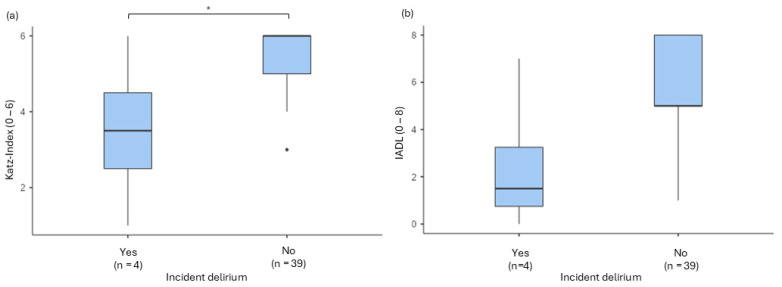
Comparison of the preoperative (**a**) Katz Index and (**b**) IADL related to delirium incidence in elective surgery (* *p* < 0.05; dots = outliers).

When comparing the preoperative mean values of Katz Index and IADL (*n* = 43) between patients with and without delirium, notable differences were observed. The IADL scores (delirium IQR 2.50, 95% CI [2.45; 7.45], no delirium IQR 3.00, 95% CI [5.26; 6.43]) showed a significant difference between the groups, U = 26.5, *p* = 0.025, and r = 0.660. However, the Katz Index scores did not show a significant difference in a robust ANOVA (*p* = 0.113; see Figure 5).

The incidence of delirium was 9.1% (*n* = 4) among elective surgery patients (*n* = 44) and 18.2% (*n* = 20) among emergency surgery patients (*n* = 110).

## 4. Discussion

The main result of this study is that impairment in ADL is significantly associated with an increased risk of postoperative delirium. The hypothesis that the incidence of postoperative delirium is influenced not only by factors such as anesthesia depth or intraoperative medications but also predominantly by the patient’s pre-hospitalization independence in daily living is confirmed by multiple parameters analyzed in this study. 

Our findings demonstrate that patients with reduced competence in performing ADL, regardless of whether they belong to the elective or emergency group, exhibit a heightened risk of postoperative delirium. The supporting evidence is provided by the significant differences in IADL scores between patients with and without postoperative delirium. Our study also identified gender-specific differences in IADL scores, with a significant correlation between low IADL scores and the occurrence of delirium in men and women. These findings suggest a potential gender-specific impact on delirium risk, warranting further investigation with larger sample sizes to confirm these trends.

We were able to expand the existing knowledge by performing a comprehensive geriatric assessment (CGA) and analyzing an adjustable predisposing risk factor: the competence in ADL. The observed correlation between functional impairment and the incidence of delirium underscores the importance of ensuring an orthogeriatric co-management (OGCM), which includes intensive therapeutic and activating nursing care, to both prevent and manage postoperative delirium. Our findings align with the recommendations of other researchers, such as Maekawa et al., advocating a preoperative geriatric assessment to screen patients according to their risk of delirium [26]. Therefore, we propose that the Katz Index and IADL can serve as surrogate markers for assessing the need for prehabilitation in patients at risk of postoperative delirium. Given that a decline in the Katz Index or IADL may predict an elevated risk of postoperative delirium, prehabilitation measures are particularly crucial. Further research with larger sample sizes and extended follow-up periods is warranted to assess whether prehabilitation positively impacts delirium incidence in at-risk patients.

Risk factors for postoperative delirium can be categorized into predisposing and precipitating factors [9,10]. Predisposing factors can increase a patient’s susceptibility to delirium regardless of the surgical procedure, while precipitating factors can trigger or exacerbate delirium depending on the type and course of surgery [11]. Daily living competence limitations represent a modifiable predisposing risk factor, and the preoperative intervention window offers an opportunity to reduce delirium incidence through prehabilitative strategies, thereby minimizing the individual risk. Considering the enhancement of preoperative daily living competence, structured outpatient exercise therapy, targeted strength training, nutritional counseling, and analysis, as well as psychological support, could provide an optimal foundation for the upcoming stress and rapid, complication-free recovery [27,28,29]. The development of a prehabilitation program through interdisciplinary collaboration between physiotherapy, gastroenterology, and psychotherapy could establish preoperative rehabilitation as an effective strategy for preventing postoperative delirium.

Based on our results, we, along with Yin et al., recommend the implementation of standardized, evidence-based non-pharmacological interventions for postoperative delirium to improve clinical care in this area [30]. For emergency patients we suggest promoting early postoperative mobilization as a means of preventing the onset of postoperative delirium in the days following surgery, thereby further reducing the associated risk. The role of intensive postoperative rehabilitation, including physiotherapy and OGCM, should be further explored regarding its protective effects on delirium incidence.

Our results indicate a delirium incidence of 9.1% among elective patients and 18.2% among emergency patients, with an overall incidence of 18.0% across all departments analyzed. These findings are consistent with those reported in the literature and contribute to the growing body of evidence that patients aged 70 and older are generally at an elevated risk for postoperative delirium. For instance, Chen et al. reported delirium incidence ranging from 24.4% to 44.9%, while Wilson et al. observed an incidence of 23% [31,32,33]. This aligns with Fuchs et al., who identified a delirium incidence of 32%, and Pazouki et al., who reported an incidence of 26% [34,35]. The slightly lower incidence in our findings may also be due to our already established OGCM.

Furthermore, our data suggest that restrictions in ADL, as measured by the Katz Index, may be less significant in the elective setting, where age, for example, emerges as a stronger risk factor. Although patients with postoperative delirium in the elective cohort tend to have lower initial Katz Index scores, the difference is not statistically significant (*p* = 0.113), implying a potential correlation between general medical condition and postoperative delirium risk. 

This study emphasizes the need for further targeted research on delirium-related health data and various predisposing factors through comprehensive geriatric assessments, as previously suggested in the literature [36,37,38]. Such research is essential for improving our understanding of delirium risk factors and enhancing our ability to detect and manage this condition effectively. In times of demographic change and the associated increasing importance of geriatric trauma centers for our healthcare system, we strongly advocate for the adoption of these simple screening tools for delirium prevention that can be integrated into everyday clinical practice. This is often not a problem of knowledge, but a problem of implementation. The responsibility here lies with all participants.

In our department, we have already firmly established the general measures described as a clinical routine for delirium prevention. Each patient receives a screening with the Nu-Desc score once per shift for three days by the appropriately qualified nursing staff. To provide structure and orientation, preventive, non-pharmacological general measures, are carried out for patients who are particularly at risk of delirium (e.g., status post delirium or cognitive impairment). Intensified OGCM is essential for this purpose.

## 5. Conclusions

We propose the implementation of a preoperative screening using the Katz Index and IADL to provide a preliminary estimation of delirium risk. We recommend promoting early postoperative mobilization, including intensive physiotherapy and orthogeriatric co-management, as an effective strategy to prevent the occurrence of postoperative delirium. For elective patients, prehabilitation should be considered when a decrease in daily living competence is identified.

## Figures and Tables

**Table 1:** Overview of the master data and output parameters.

Gender	Emergency (*n* = 111)	Elective (*n* = 46)
**Men**	33.3% (*n* = 37)	21,7% (*n* = 10)
**Women**	66.7% (*n* = 74)	78,2% (*n* = 36)
Age ***	83.0 ± 6.76	80.0 ± 5.46
**Height**	167.0 ± 8.62 cm	167 ± 9.24 cm
Weight **	68.0 ± 15.4 kg	74.5 ± 16.0 kg
Body-Mass-Index ***	23.0 ± 4.56	26.5 ± 5.40
Underweight	2.5% (*n* = 3)	0.0% (*n* = 0)
Normal weight	59.3% (*n* = 64)	36.4% (*n* = 16)
Overweight	22.2% (*n* = 24)	52.3% (*n* = 23)
Obesity	15.7% (*n* = 17)	11.4% (*n* = 5)
Mini-Nutritional-Assessment ***	*n* = 100 9.29 ± 2.41	*n* = 42 11.2 ± 2.53
Normal nutritional status (12–14)	27.0% (*n* = 27)	59.5% (*n* = 25)
Risk for malnutrition (8–11)	47.0% (*n* = 47)	26.2% (*n* = 11)
Malnutrition (0–7)	26.0% (*n* = 26)	14.4% (*n* = 6)

Significant differences (** *p* < 0.01; *** *p* < 0.001) between emergency and elective patients were observed in age (t (155) = −4.03, *p* < 0.001), weight (U = 1766, *p* = 0.011, r = 0.26), Body Mass Index (U = 1543, *p* < 0.001, r = 0.35), and Mini Nutritional Assessment (U = 1156, *p* < 0.001, r = 0.45).

**Table 2:** Overview Model Coefficients: models for predictability of the preoperative parameters (Katz Index, IADL) of incidence of delirium in emergency surgery. Note. The cut-off value is set to 0.5.

Model Fit Measures							
					Overall Model Test		
Model	Deviance	AIC	R^2^_McF_	R^2^_N_	χ^2^	df	*p*
1	84.8	90.8	0.132	0.193	12.9	2	0.002
**Model Coefficients—Delirium yes/no**							
						**95% Confidence Interval**	
**Predictor**	**Estimate**	**SE**	**Z**	***p***	**Odds ratio**	**Lower**	**Upper**
**Intercept**	−0.658	0.775	−0.848	0.396	0.518	0.113	2.37
**Katz Index**	0.199	0.217	0.914	0.361	1.22	0.797	1.87
**IADL**	0.302	0.162	1.863	0.062	1.353	0.984	1.86

Note. Estimates represent the log odds of “Delirium yes/no = no” vs. “Delirium yes/no = yes”.

**Table 3:** Overview Model Coefficients: Models for predictability of the preoperative parameters (Katz Index, IADL) of incidence of delirium in elective surgery. Note. The cut-off value is set to 0.5.

Model Fit Measures							
					Overall Model Test		
Model	Deviance	AIC	R^2^_McF_	R^2^_N_	χ^2^	df	*p*
1	16.5	22.5	0.379	0.453	10.1	2	0.006
**Model Coefficients—Delirium yes/no**							
						**95% Confidence Interval**	
**Predictor**	**Estimate**	**SE**	**Z**	***p***	**Odds ratio**	**Lower**	**Upper**
Intercept	−3.432	2.573	−1.334	0.182	0.0323	2.09 × 10^−4^	5.01
Katz Index	0.827	0.693	1.194	0.232	2.2872	0.588	8.89
IADL	0.403	0.414	0.973	0.331	1.4957	0.664	3.37

Note. Estimates represent the log odds of “Delirium yes/no = no” vs. “Delirium yes/no = yes”.

## Data Availability

The data presented in this study are available on request. For further information please contact the corresponding author.
